# Exploring Tissue Transglutaminase Antibodies as a Confirmatory Tool for Diagnosing Coeliac Disease in Children

**DOI:** 10.3390/diagnostics16081216

**Published:** 2026-04-19

**Authors:** Petra Riznik, Patricija Levstik, Mojca Kukovic, Jernej Dolinsek

**Affiliations:** 1 Department of Pediatrics, Gastroenterology, Hepatology and Nutrition Unit, University Medical Centre Maribor, 2000 Maribor, Slovenia; 2 Faculty of Medicine, University of Maribor, 2000 Maribor, Slovenia

**Keywords:** coeliac disease, children, diagnosis, no-biopsy approach, tissue transglutaminase antibodies, anti-endomysial antibodies, duodenal biopsy

## Abstract

**Background/Objectives:** The prerequisite for diagnosing celiac disease (CD) in children without duodenal biopsy is a tissue transglutaminase antibody (TGA) level exceeding 10 times the upper limit of normal levels, along with a positive confirmatory anti-endomysial antibody (EMA) test in a secondary blood sample. The aim of our study was to determine whether determination of TGA in a second blood sample could be as reliable as determination of EMA as the confirmatory test in children eligible for the no-biopsy approach. **Methods:** A retrospective analysis of medical data was conducted, including children under 19 years old who were diagnosed with coeliac disease at our department from January 2013 to June 2025. We examined the diagnostic process, focusing on TGA levels at the time of diagnosis. **Results:** Data from 185 CD patients (59.5% female, median age 8 years) were available for the analysis. 49 (26.5%) patients were diagnosed using no-biopsy approach and 46 (93.9%) of those had TGA > 10 × ULN in second blood sample as well. In the group of children diagnosed using duodenal biopsy (*N* = 136; 96.3% 3 Marsh), 78 (57.4%) children had initial TGA > 10 × ULN. In 34 (43.6%) of those children, secondary serology performed before introducing a diet showed that 30 (88.2%) of them had TGA > 10 × ULN. **Conclusions:** Our study suggests that TGA levels > 10 × ULN could serve as a reliable test for confirming the diagnosis of CD in children, eligible for the no-biopsy approach, thus making the diagnostic process more cost-effective and efficient, while maintaining accuracy.

## 1. Introduction

Coeliac disease (CD) is a chronic, immune-mediated enteropathy triggered by gluten ingestion in genetically susceptible individuals. It affects approximately 1% of the population worldwide, although many cases remain undiagnosed due to heterogeneous clinical presentations and variable diagnostic practices [1,2,3,4]. The clinical manifestations of coeliac disease can be attributed to a combination of intestinal inflammation, nutrient deficiencies resulting from malabsorption, and the autoimmune response directed against tissue transglutaminase. Historically, coeliac disease was considered a disorder of childhood, typically presenting with chronic diarrhoea and features of malabsorption. However, it is now recognized as a systemic disease that can occur at any age. Although impaired intestinal absorption is a hallmark of the disease, gastrointestinal symptoms are present in only approximately half of patients. This is because coeliac disease is not confined to the gastrointestinal tract and may present with a wide range of intestinal and extraintestinal manifestations or even remain asymptomatic. Extraintestinal manifestations can involve multiple organ systems, including the nervous system, liver, skin, reproductive system, cardiovascular system, and musculoskeletal system. These manifestations are often associated with more severe clinical and histological disease. Furthermore, while some may present in early childhood, others may not become apparent until adulthood or later in life [1,2,5]. Early identification and treatment are essential to prevent long-term complications such as osteoporosis, infertility, and intestinal malignancy [6]. Traditionally, the diagnosis of CD has relied on the detection of disease-specific serum antibodies, principally anti-endomysial antibodies (EMA) and anti-tissue transglutaminase antibodies (TGA) in combination with histological evaluation of small intestinal biopsies [7]. The identification of the EMA IgA assay in 1983 marked the beginning of modern serological testing for CD, representing a major advance due to its superior sensitivity and specificity compared with earlier methods. EMA assays are mostly based on indirect immunofluorescence using monkey oesophagus or human umbilical cord as the substrate. Although this technique is relatively standardized, it remains subjective and is characterized by notable inter- and intra-observer variability [8]. Nonetheless, in laboratories with substantial expertise, EMA continues to offer the highest positive predictive value for coeliac disease [8]. In 1997, tissue transglutaminase was identified as the coeliac autoantigen. This pivotal discovery enabled the development of ELISA-based tests that overcame many of the limitations associated with interpreting EMA immunofluorescence patterns. Early tissue transglutaminase assays, however, were affected by high false-positive rates due to impurities in the guinea pig liver tissue transglutaminase used as antigen in commercial kits. The subsequent introduction of human recombinant tissue transglutaminase markedly improved the diagnostic accuracy of the ELISA test [8]. The evolution of European Society for Pediatric Gastroenterology, Hepatology and Nutrition (ESPGHAN) diagnostic criteria (1969 to 2020) reflects these advances. The first formalized criteria, the Interlaken criteria of 1969, required at least three duodenal biopsies—initial biopsy on a gluten-containing diet, the second after introduction of a gluten-free diet, and the third following a gluten challenge—and served as the international standard for over 20 years [9]. The 1990 revision removed the need for gluten challenge in children over 2 years and incorporated serological testing, with a single biopsy plus clinical and serological improvement after a gluten-free diet being sufficient for diagnosis [10]. The 2012 ESPGHAN guidelines introduced a “no-biopsy” diagnostic option for children meeting specific criteria: symptomatic presentation, TGA levels higher than 10× upper limit of normal (ULN), a positive EMA test on a second blood sample, as well as the presence of HLA DQ2 or DQ8, along with the consent from the patient and/or caregiver [11]. Most recent ESPGHAN guidelines, updated in 2020, allow for the no-biopsy approach for all children with initial TGA > 10 × ULN and positive confirmatory EMA, regardless of symptoms, and without the need for genetic testing. Diagnosis must be established by a paediatric gastroenterologist, and patient and/or caregiver consent remains mandatory [2].

Although the no-biopsy approach has been used for diagnosing CD in children for the past 13 years, adult gastroenterologists have mostly remained reluctant to adopt this strategy. The first guidelines allowing the no-biopsy approach in adults were introduced in Finland. Ylönen et al. [12] showed that serology-based diagnosis using various commercial assays and pre-test probabilities is highly accurate in adults when TGA levels are ≥10 × ULN. Likewise, in India, Pachisia et al. [13] reported that approximately half of adult patients with CD could safely benefit from the no-biopsy pathway, thereby reducing healthcare burden and minimizing the risks associated with gastroscopy and anaesthesia. Cauci et al. [14] also supported the biopsy-free strategy in adults with TGA ≥ 10 × ULN, particularly in the presence of positive EMA and typical clinical presentation. A metanalysis by Shiha et al. [15] further corroborated these findings, indicating that selected adult patients with TGA ≥ 10 × ULN and a moderate-to-high pre-test probability of CD can be reliably diagnosed without invasive endoscopy or duodenal biopsy. Most recently, the European Society for the Study of Coeliac Disease has endorsed the no-biopsy approach in adults under 45 years of age with IgA TGA ≥ 10 × ULN, provided that serology is confirmed on a second blood sample [16].

However, several concerns regarding the no-biopsy approach have also been raised. One issue is the lack of standardization in serological testing, which can complicate serology-based diagnosis. This was highlighted in the North American Society for Pediatric Gastroenterology, Hepatology and Nutrition (NASPGHAN) guidelines for paediatric CD [17]. Similar concerns were reported by Alex et al. [18], who demonstrated substantial variability in coeliac serology results across laboratories in the United Kingdom, underscoring a significant barrier to adopting the no-biopsy strategy in routine adult clinical practice. Nonetheless, more recent guidelines from the American College of Gastroenterology have begun to allow a no-biopsy diagnosis in children under specific conditions [19].

Traditionally, when diagnosing CD using the no-biopsy approach, EMA has been used as the confirmatory test in children with high TGA levels. With the increasing reliability, standardization, and availability of TGA assays, it is important to examine whether TGA alone could serve as a confirmatory marker and streamline the diagnostic process. Therefore, the aim of our study was to assess whether determination of TGA in a second blood sample could be as reliable as determination of EMA as the confirmatory test in children eligible for the no-biopsy diagnosis.

## 2. Methods

We conducted a single centre retrospective study, including children and adolescents under 19 years of age, who were diagnosed with coeliac disease and managed at the Department of Paediatrics, Gastroenterology, Hepatology and Nutrition Unit, University Medical Centre Maribor, Slovenia, from January 2013 to June 2025. Data were retrieved from electronic medical records and included age at diagnosis, clinical presentation, CD serology results (TGA and EMA) and histopathological findings when performed. The exclusion criteria were selective IgA deficiency (hypogammaglobulinemia A), incomplete serological or histopathological data, and patients whose diagnosis was established outside our centre. Serum TGA-IgA levels (Eurospital, Trieste, Italy, or Thermo Fisher, Waltham, MA, USA) and EMA-IgA (Eurospital, Trieste, Italy or Thermo Fisher, Waltham, MA, USA) were measured at the Department of Laboratory Diagnostics. Biopsy specimens were evaluated and classified according to the Marsh–Oberhuber classification.

The diagnosis of CD was established according to the ESPGHAN guidelines valid at the time of diagnosis [2,11]. Children were diagnosed based on either a no-biopsy approach, defined as TGA levels > 10 × ULN with positive EMA in a second independently obtained blood sample, and confirmation by a paediatric gastroenterologist with caregiver consent; or by histological confirmation via duodenal biopsy in patients who did not meet the serological criteria for a no-biopsy diagnosis, or in whom biopsy was clinically indicated according to local practice at the time. All diagnoses were established in a paediatric gastroenterology setting following standard clinical protocols. In our centre, the implementation of the no-biopsy approach evolved over time. In the earlier years of the study period (2013–2020), duodenal biopsy was performed more frequently, even in some cases where no-biopsy criteria were met, due to institutional practice at the time, ongoing research protocols, and shared decision-making with parents. In later years (2021–2025), in line with increasing adoption of the ESPGHAN recommendations across Europe, the no-biopsy approach was more consistently applied in eligible patients, always following guideline criteria and after discussion with parents.

Patients were categorized based on whether they underwent duodenal biopsy or were diagnosed using no-biopsy approach. In the no-biopsy group, two serological samples were obtained, with the second serving as a confirmatory test; TGA were measured along with EMA in the second blood sample. We wanted to determine whether patients with an initial TGA > 10 × ULN and positive EMA in the confirmatory sample also demonstrated persistently elevated TGA levels in that same sample. For patients who underwent duodenal biopsy, additional CD serological tests (TGA and EMA) were performed at the time of the biopsy.

Statistical analyses were performed using IBM SPSS Statistics, version 22.0. Categorical variables were analysed using the Chi-square test. Comparisons of continuous variables were performed using Student’s *t*-test for normally distributed data and the Mann–Whitney U test for non-normally distributed data. One-way ANOVA was used for comparisons across multiple groups. A *p*-value < 0.05 was considered statistically significant.

The study was approved by the National Medical Ethics Committee of the Republic of Slovenia 0120-405/2024-2711-4.

## 3. Results

**Figure 1:**
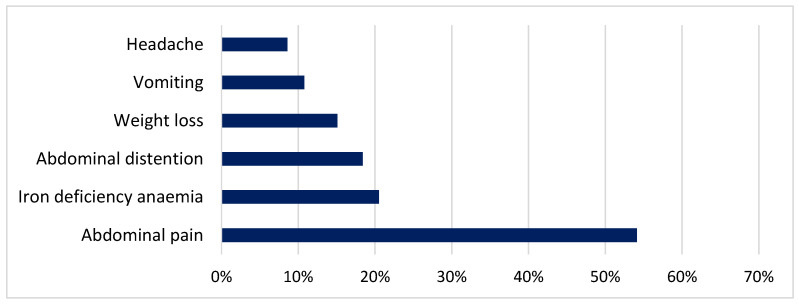
Most common symptoms and signs of CD.

Data from 185 children diagnosed with CD (59.5% female, median age 8 years) were included in the study. Of these, 168 children (90.8%) were symptomatic at diagnosis, while the remaining 17 were identified through risk-group screening (mostly positive family history of CD). A positive family history of CD was reported in 52 cases (28.1%) overall. Among symptomatic children, the most frequently reported symptoms and signs were abdominal pain (54.1%), iron deficiency anaemia (20.5%) and abdominal distention (18.4%) (Figure 1).

**Figure 2:**
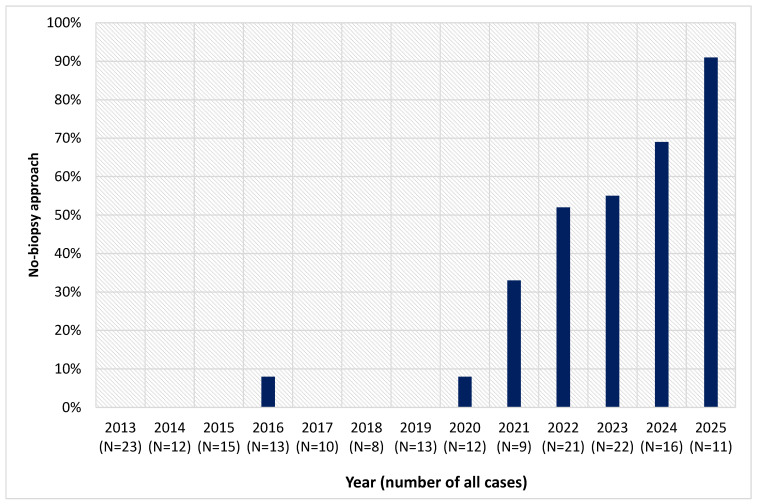
Year-to-year changes in number of children diagnosed with CD using the no-biopsy approach at our centre compared to overall number of newly diagnosed patients.

In 136 children (73.5%; median age 7 years) diagnosis was confirmed using duodenal biopsy, with Marsh 3 lesion detected in 96.3% of children. Remaining 49 children (26.5%; median age 9 years) were diagnosed using the no-biopsy approach (Table 1). The no-biopsy approach has been used more often after 2021 (47 children 2021–2025 vs. 2 children 2013–2020; *p* < 0.001; Figure 2).

Among children undergoing biopsy, 78 (57.4%) had initial TGA levels > 10 × ULN, yet still proceeded to biopsy. In 34 of these (43.6%), repeat serological testing (TGA and EMA) was performed at the time of biopsy and in 30 children TGA levels exceeded 10 × ULN in this second sample. Among the remaining four children, three had already been partially adhering to a gluten-free diet prior to biopsy (TGA > 5 × ULN), and one had a TGA level of 6 × ULN.

Of the 58 children with an initial TGA level < 10 × ULN, repeat serological testing was performed in 40 (68.9%). In four of these children, TGA increased to >10 × ULN, while in the remaining 36 it remained <10 × ULN.

**Figure 3:**
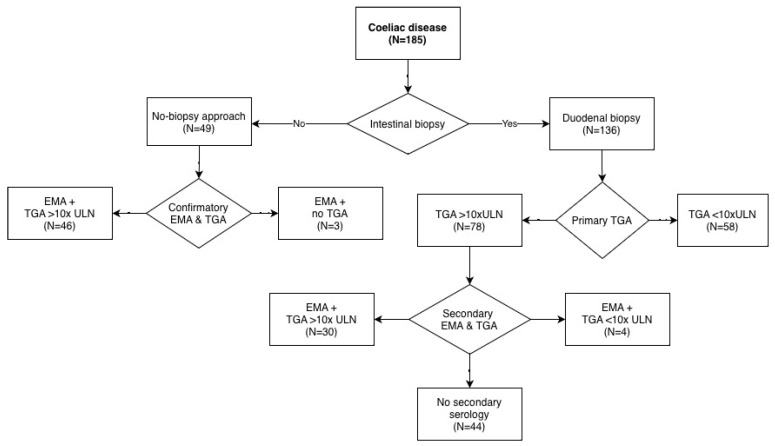
CD diagnostic flowchart.

In the group diagnosed using the no-biopsy approach, all children had initial TGA levels > 10 × ULN. In 46 of 49 children (93.9%), repeat TGA testing was performed alongside EMA as a confirmatory test, and all maintained TGA levels > 10 × ULN. In the remaining three children, only EMA was obtained as confirmatory testing (Figure 3). The median interval between the initial and confirmatory samples in the no-biopsy group was 21 days, significantly shorter than in children undergoing duodenal biopsy (94 days; *p* < 0.001).

Among symptomatic children (*N* = 168), 127 (75.6%) were diagnosed using duodenal biopsy, whereas among asymptomatic children (*N* = 17), 9 (53%) were diagnosed using biopsy. However, according to the ESPGHAN guidelines, no-biopsy diagnosis in asymptomatic children was possible since 2020, leading to a lower number of no-biopsy diagnosis in this group despite the fact that most asymptomatic children (88.2%) had TGA > 10 × ULN. In children with TGA > 10 × ULN (*N* = 127), abdominal pain was reported as the primary symptom in 48.8%, compared with 65.5% in the group of children with initial TGA < 10 × ULN. Nevertheless, the three most common symptoms were the same between children diagnosed with the biopsy and no-biopsy approaches. Median age of children with TGA > 10 × ULN was lower compared to those with TGA < 10 × ULN (7 years vs. 10 years; NS). Among children with an affected first-degree family member (*N* = 52), 78.9% had TGA > 10 × ULN, compared with 64.7% among those without a family history of coeliac disease (86 out of 133 children had TGA > 10 × ULN). Also, there was a significantly higher proportion of asymptomatic children (16 out of 52 children) in the group of children with a family member having CD compared to the group of children without a family member with CD (1 out of 133 children; 30.8% vs. 0.8%; *p* < 0.001).

## 4. Discussion

We found that all children diagnosed with coeliac disease using the no-biopsy approach still had very high TGA levels when confirmatory serology was performed. All children diagnosed with coeliac disease since 2013, when the first guidelines allowing the no-biopsy approach for diagnosing coeliac disease in symptomatic children were introduced, were included in the study. However, intestinal biopsy was still used very frequently during the early years after the introduction of the no-biopsy approach, mostly due to the ongoing research activities in the field of coeliac disease in which our centre was involved. In almost all children who had high TGA levels prior to biopsy and were subsequently confirmed to have coeliac disease, TGA levels remained markedly elevated—more than 10 × ULN when serological testing was repeated at the time of biopsy. Only in four children had TGA levels fallen below 10 × ULN, mostly because the family had already initiated a gluten-free diet contrary to our instructions. Similar findings were reported in the study by Ben-Tov et al. [20]. Among 933 patients who had high-positive TGA levels > 10 × ULN in their initial sample, all demonstrated both high-positive TGA and positive EMA on confirmatory testing. The authors therefore suggested that a repeated TGA test could replace EMA as the confirmatory serological test in the no-biopsy diagnostic approach for coeliac disease [20]. In the Joint British Society for Pediatric Gastroenterology, Hepatology and Nutrition and Coeliac UK guidelines for the diagnosis and management of coeliac disease in children [21], published in 2013, the use of TGA as a substitute for EMA was already permitted in situations where EMA testing was not locally available. Several other studies have also evaluated the proposed no-biopsy approach. Pacheco et al. [22] demonstrated that TGA has higher sensitivity and greater specificity and positive predictive value at ≥10 × ULN compared with EMA, indicating that TGA ≥ 10 × ULN is superior to EMA for the serologic diagnosis of coeliac disease [22]. Thompson et al. [23] likewise found that adding HLA typing and EMA testing in patients with high-titre TGA does not improve diagnostic performance and may be omitted from the serological workup in these cases [23]. Similar findings were reported by Anat Guz-Mark et al. [24], who additionally showed that a multiplex TGA assay had very high diagnostic accuracy in real-life settings; among patients with positive TGA, the addition of EMA did not enhance diagnostic performance. Furthermore, false-positive rates varied across different TGA ranges but remained low when TGA levels exceeded 10 × ULN [24].

Although EMA testing remains highly specific for coeliac disease, it is used less frequently in routine practice because it is more expensive, labour-intensive, and technically demanding than automated ELISA-based assays. In the study by Pumar et al. [25], it was shown that the combination of TGA ≥ 10 × ULN and DGP IgG ≥ 3.5 × ULN provided a high positive predictive value for coeliac disease and could potentially replace EMA testing in children with TGA ≥ 10 × ULN. However, in their study, TGA and DGP were performed as paired tests from a single blood draw, and no direct comparison between EMA and DGP was possible because EMA testing was not available at their institution. Moreover, children diagnosed using the no-biopsy approach were excluded from their analysis [25]. Similarly, Rispo et al. [26] demonstrated that the combined TGA/DGP approach is accurate for the diagnosis of coeliac disease and could reduce both the costs and operator-dependency associated with EMA, suggesting that DGP together with TGA may replace EMA in the diagnostic pathway [26].

In our study, children diagnosed using the no-biopsy approach were older than those diagnosed by duodenal biopsy. This finding contrasts with the results of Trovato et al. [27], who reported that patients with markedly elevated antibody titres (≥10 × ULN) were diagnosed at a younger age than those with lower titres. We also showed that the diagnosis of coeliac disease was established somewhat faster in children diagnosed using the no-biopsy approach. This was mainly because obtaining a second blood sample for confirmatory serology required significantly less time than scheduling and performing a duodenal biopsy. Biopsy procedures had longer waiting times due to the need for general anaesthesia and the requirement that children be free of respiratory infections at the time of endoscopy, both of which contributed to delays. Another important factor could also be the reluctance of parents to submit their child to a biopsy and therefore later appointment for the procedure. Furthermore, in children with low serology at presentation, biopsies were often intentionally scheduled later to assure that a child was exposed to sufficient amounts of gluten and therefore mucosal changes were more likely, increasing the likelihood of confirming coeliac disease through histopathological evaluation. We also found it interesting that children with a family history of coeliac disease more frequently presented with high TGA levels compared with those without affected relatives. At our institution, children from this risk group undergo annual testing for coeliac disease, which usually minimizes diagnostic delay, especially when they become symptomatic. It is of interest that many of these children are potentially exposed to less gluten at home since they consume a partially gluten-free diet because another family member has coeliac disease; therefore, one would intuitively expect these children to have lower titres of celiac disease specific serology.

Our study has several important limitations. First, it is a retrospective study; therefore, we were unable to obtain all desired data for the included patients, and no predefined study protocol was available. As a result, a second serological sample was not obtained in the majority of children diagnosed by duodenal biopsy, as this was not required according to the diagnostic guidelines at the time. However, such data would have further strengthened the study.

In addition, this is a single-centre study; therefore, the results may not be fully generalizable to other settings, particularly where different laboratory assays or diagnostic equipment are used.

Another limitation is that the no-biopsy approach was not applied in all eligible patients during the early study period due to centre-specific factors, including ongoing research activities. Consequently, the number of no-biopsy diagnoses is likely underestimated in the early years of the study.

To conclude, our study suggests that TGA levels > 10 × ULN may serve as a reliable test for confirming the diagnosis of coeliac disease in children eligible for the no-biopsy approach. Omitting EMA from the diagnostic algorithm could simplify the diagnostic process by reducing the need for specialized, labour-intensive testing and the subjectivity associated with immunofluorescence interpretation. Also, it could lower costs as automated TGA assays are less expensive, faster, and easier to standardize than EMA. Importantly, our findings indicate that relying on TGA alone in these high-titre cases does not compromise diagnostic accuracy, supporting a safe and efficient approach to coeliac disease diagnosis in children.

## Figures and Tables

**Table 1:** Characteristics of children according to the diagnostic approach.

	Duodenal Biopsy (*N* = 136)	No-Biopsy Approach (*N* = 49)	Sig.
Sex	58.8% female	61.2% female	*p* = 0.769
Age (median)	7 years	9 years	*p* = 0.293
Symptomatic	93.4%	83.7%	*p* = 0.044
TGA > 10 × ULN	57.4%	100%	N/A
CD in family	24.3%	38.8%	*p* = 0.053

## Data Availability

The data presented in this study are available on request from the corresponding author due to privacy and ethical restrictions.
